# Supplementary material for: The Intramolecular Charge Transfer Mechanism by Which Chiral Self-Assembled H8-BINOL Vesicles Enantioselectively Recognize Amino Alcohols
Source: Int J Mol Sci. 2024 May 21;25(11):5606. doi: 10.3390/ijms25115606 (PMC11171953; doi:10.3390/ijms25115606)
Supplement: Supplementary file 1 [file ijms-25-05606-s001.zip › ijms-2982114-supplementary.pdf]

Supporting Information

The Intramolecular Charge Transfer Mechanism by Which Chiral Self-assembled H_8-BINOL Vesicles Enantioselectively Recognize Amino Alcohols

Contents

1. Synthesis of RS-4 2
2. ^{1}H NMR and ^{13}C NMR 2
    2.1 ^{1}H NMR and ^{13}C NMR of R-**3** 2
    2.2 ^{1}H NMR and ^{13}C NMR of R-**2** 3
    2.3 ^{1}H NMR and ^{13}C NMR of R-**1** 5
    2.4 ^{1}H NMR and ^{13}C NMR of S-**3** 6
    2.5 ^{1}H NMR and ^{13}C NMR of S-**2** 7
    2.6 ^{1}H NMR and ^{13}C NMR of S-**1** 8
    2.7 ^{1}H NMR of RS-**4** 9
3. Fluorescence spectras of R-**2** 10
4. Comparison of IR, UV and fluorescence calculated data of R-**1** with experimental data 14
5. Specific data for electron-hole analysis 16

1. Synthesis of RS-4

According to Scheme 2 in the text, R-**1** (0.2g 0.37mmol) was added to a round-bottomed flask and S-2-amino-1-phenylethanol (0.13g 0.94mol) and 1-2 drops of glacial acetic acid were added under Ar protection and the reaction was carried out at reflux for 8 h in an ethanol system. After the reaction, a solid was precipitated, filtered and washed three times with anhydrous ethanol and hexane, and dried to give give 250 mg of RS-**4** as a yellow solid with a yield of 86%. ^{1}H NMR (400 MHz, DMSO-*d6*) δ 13.47 (s, 2H), 8.52 (s, 2H), 7.55 (s, 2H), 7.49 (d, J = 8.5 Hz, 2H), 7.41 (s, 4H), 7.34 (s, 4H), 7.26 (d, J = 7.2 Hz, 2H), 6.99 – 6.81 (m, 4H), 4.87 (s, 2H), 4.37 (s, 1H), 3.85 (s, 2H), 3.73 (s, 2H), 3.17 (s, 1H), 2.71 (s, 4H), 2.24 (d, J = 17.4 Hz, 2H), 2.01 (d, J = 17.1 Hz, 2H), 1.62 (s, 8H).

2. ^{1}H NMR and ^{13}C NMR

2.1 ^{1}H NMR and ^{13}C NMR of R-3

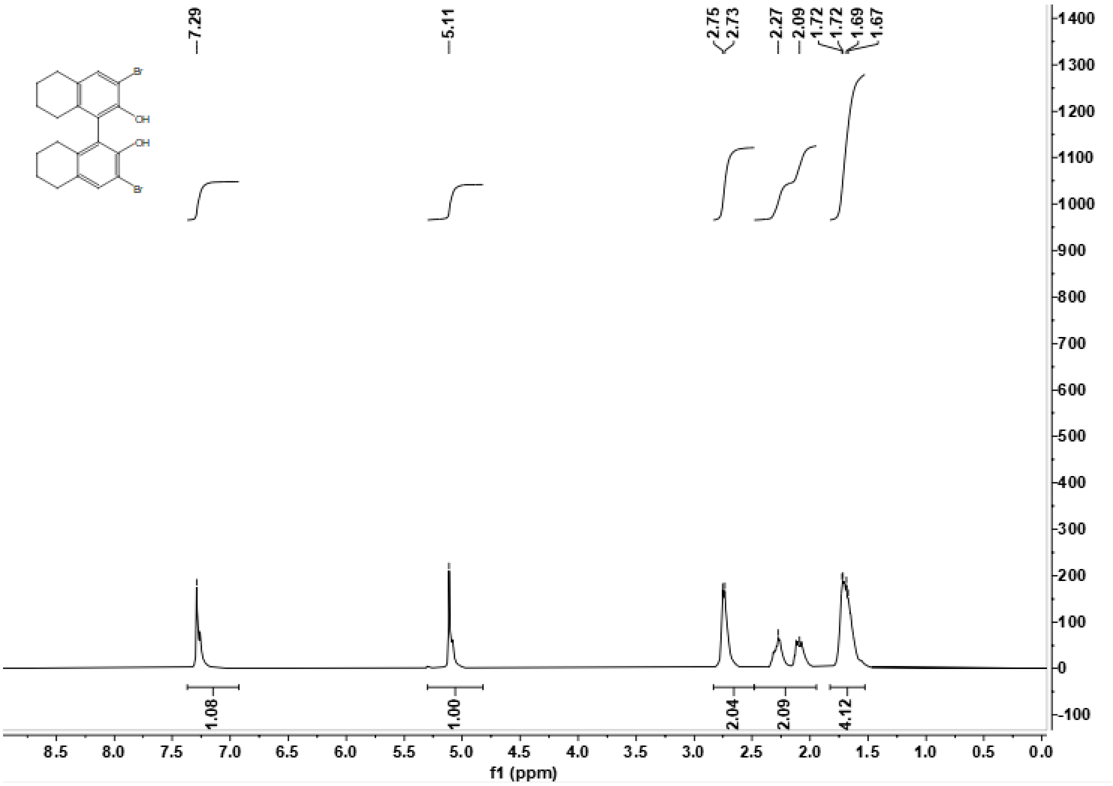

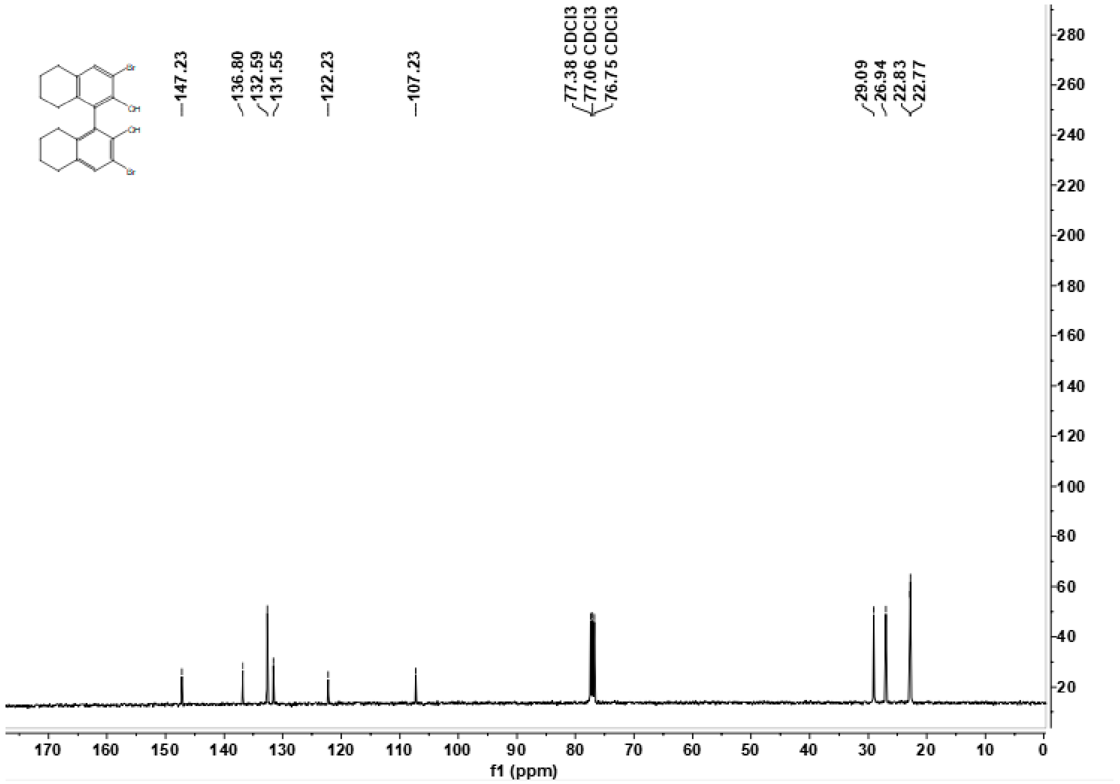

Figure S1. ^{1}H NMR, ^{13}C NMR of R-**3**($CDCl_3$)

2.2 ^{1}H NMR and ^{13}C NMR of R-2

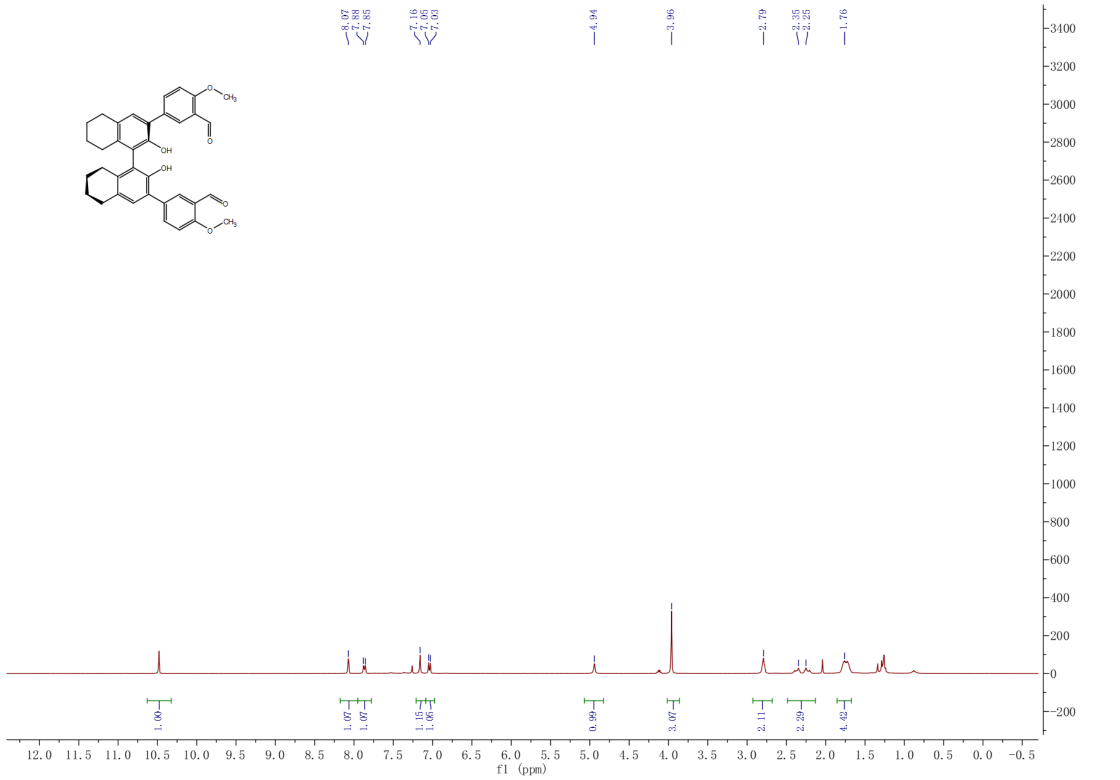

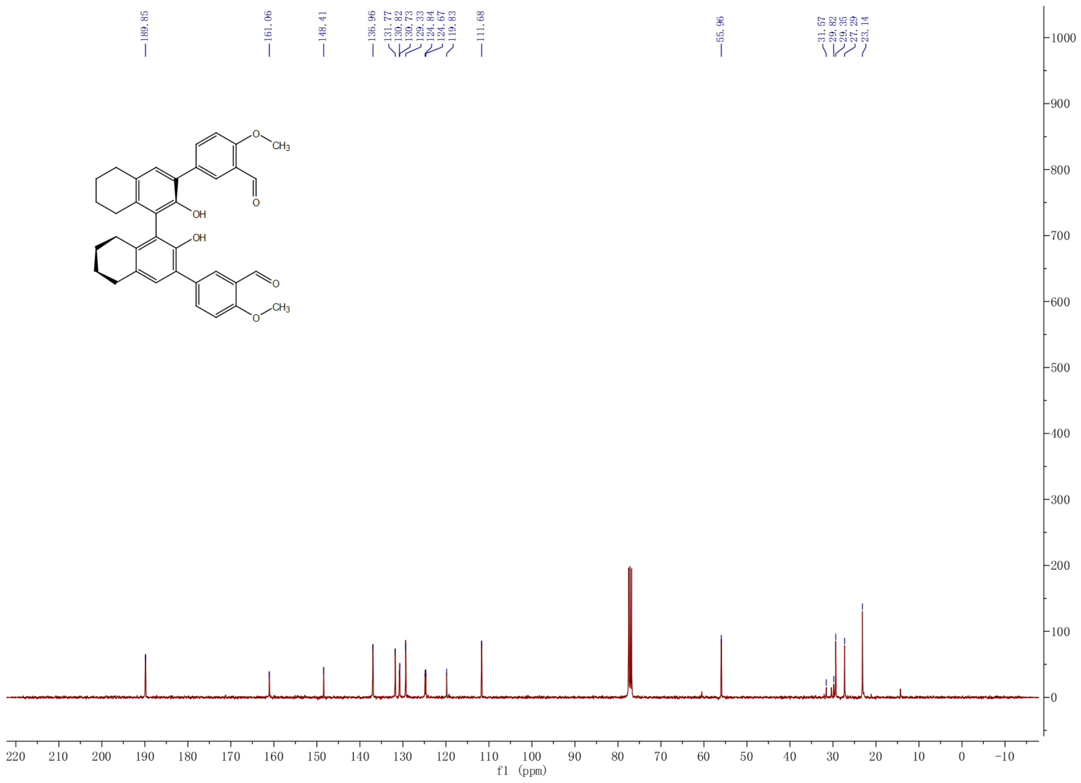

Figure S2. ^{1}H NMR, ^{13}C NMR of R-**2** ($CDCl_3$)

2.3 ^{1}H NMR and ^{13}C NMR of R-1

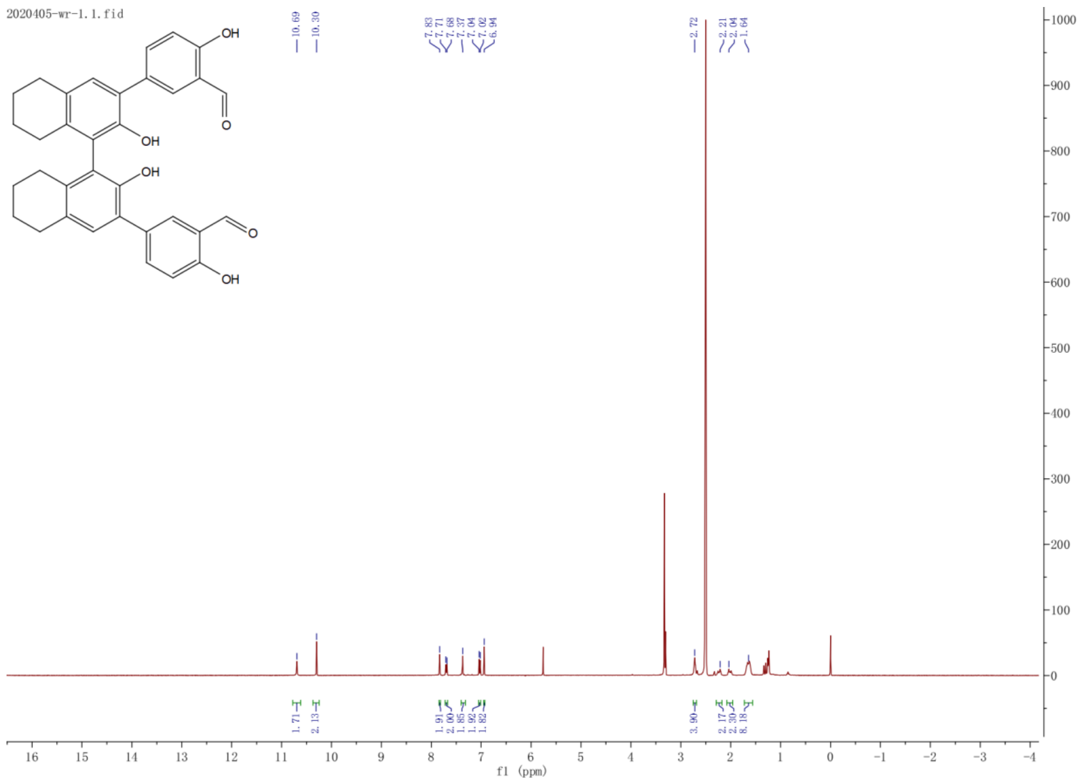

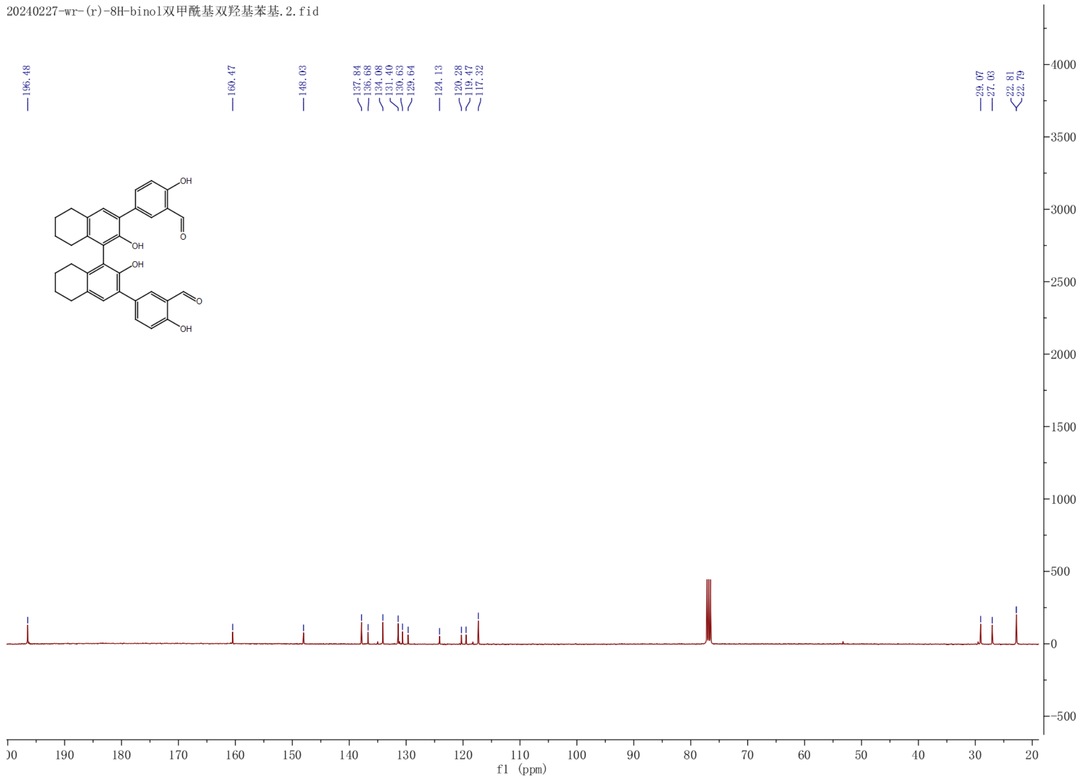

Figure S3. ^{1}H NMR of R-**1** (DMSO-*d6*), ^{13}C NMR of R-**1** ($CDCl_3$)

2.4 ^{1}H NMR and ^{13}C NMR of S-3

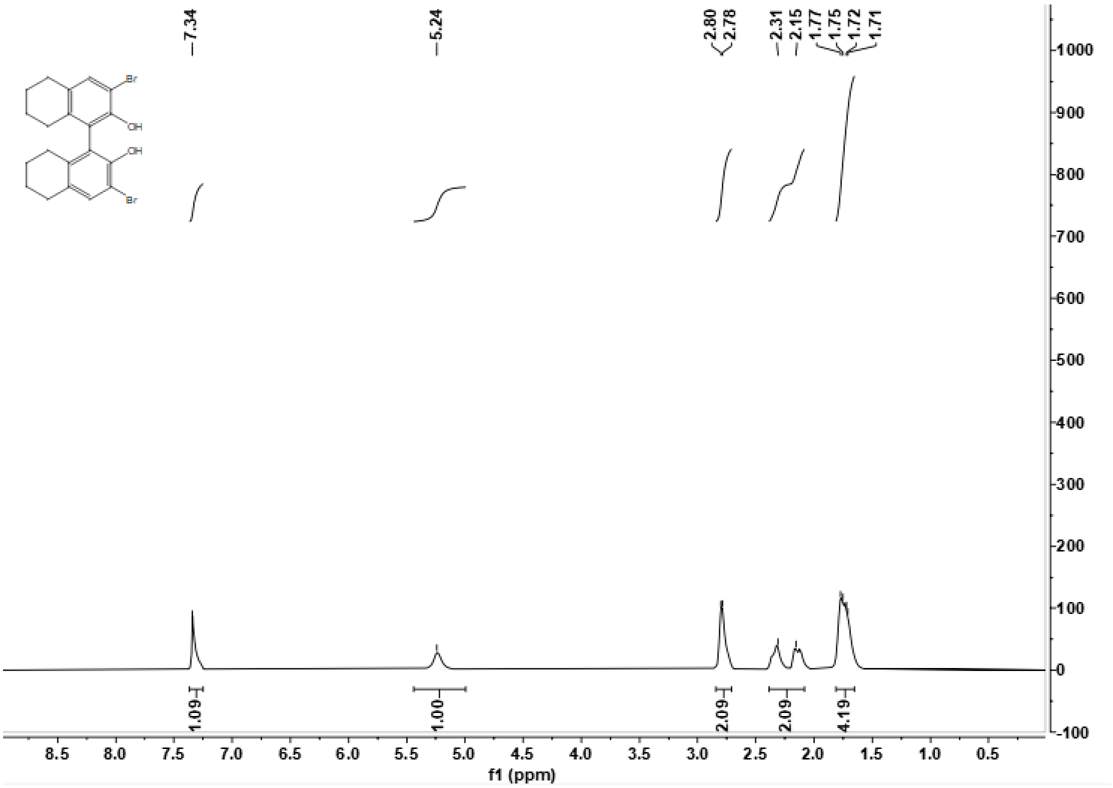

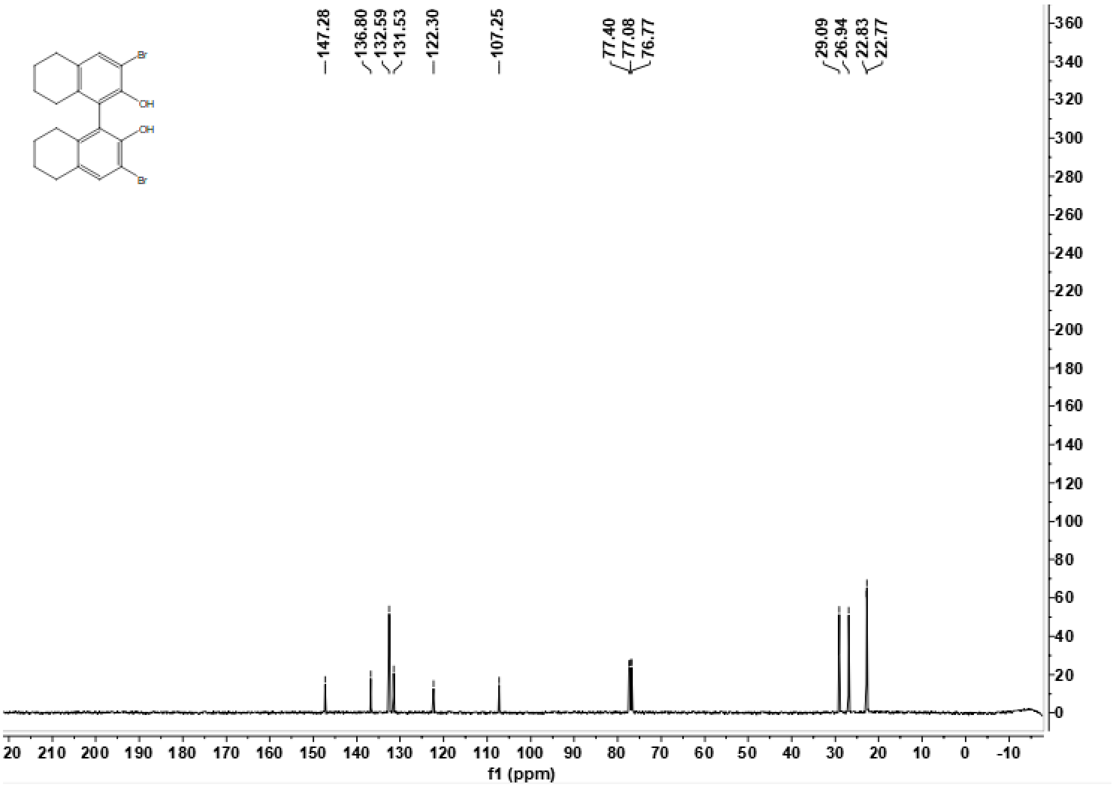

Figure S4. ^{1}H NMR, ^{13}C NMR of S-**3** ($CDCl_3$)

2.5 ^{1}H NMR and ^{13}C NMR of S-2

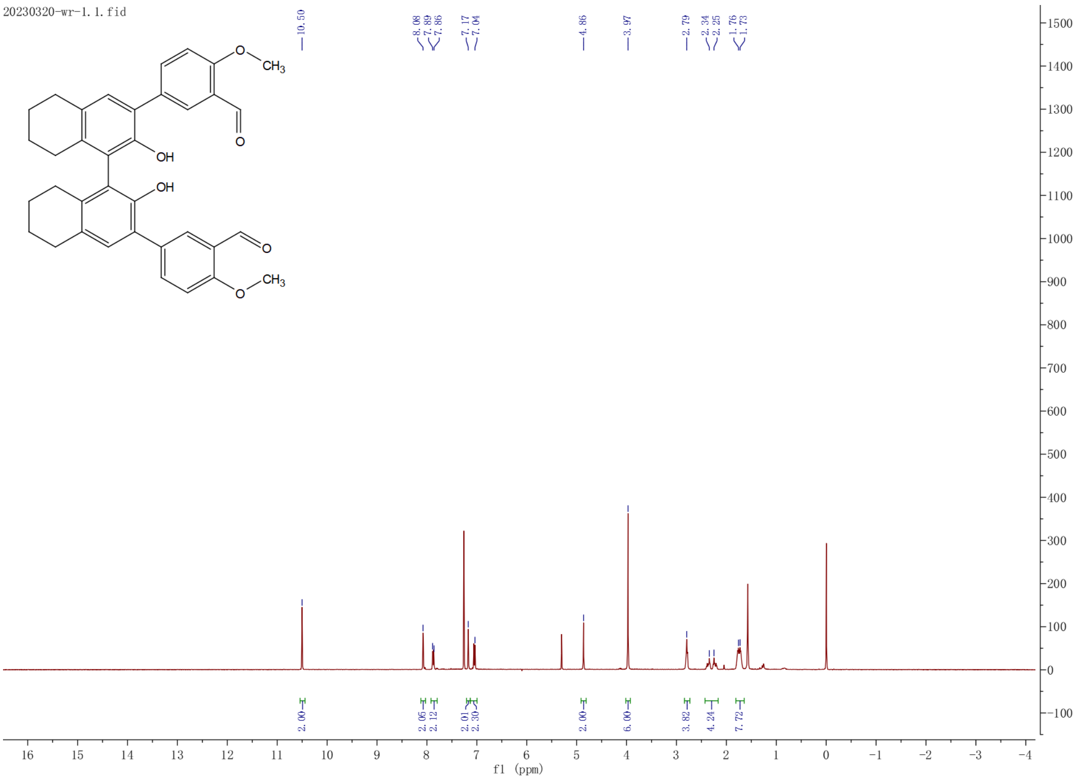

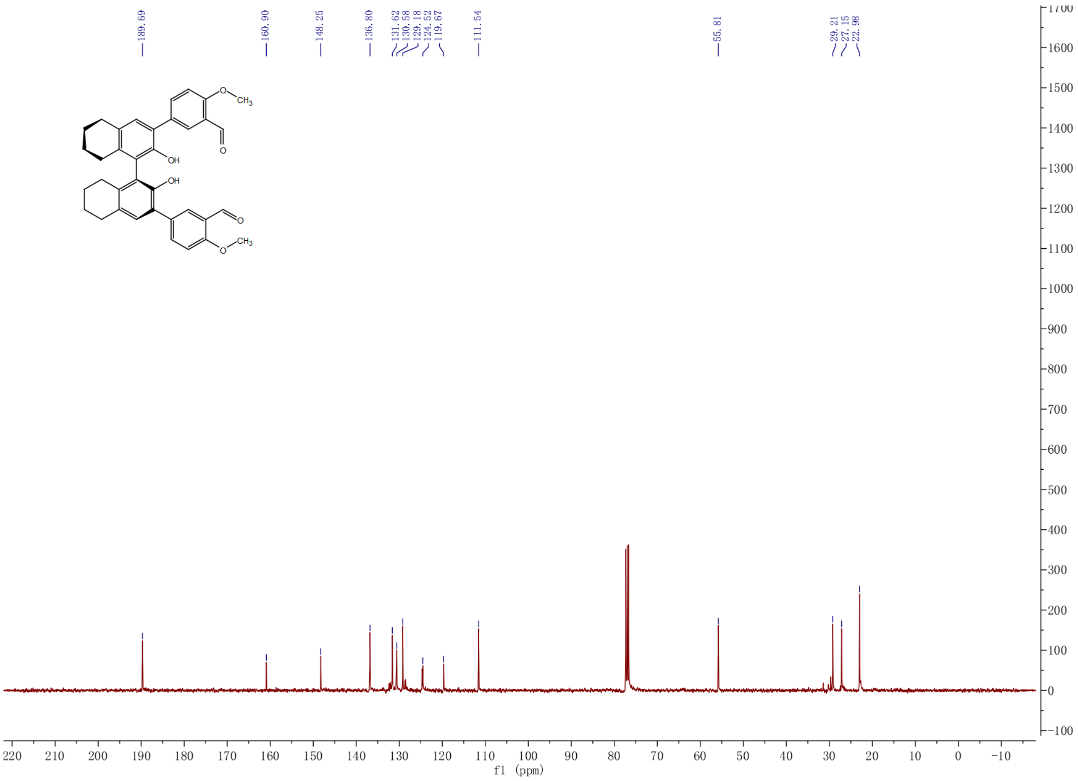

Figure S5. ^{1}H NMR, ^{13}C NMR of S-**2** ($CDCl_3$)

2.6 ^{1}H NMR and ^{13}C NMR of S-1

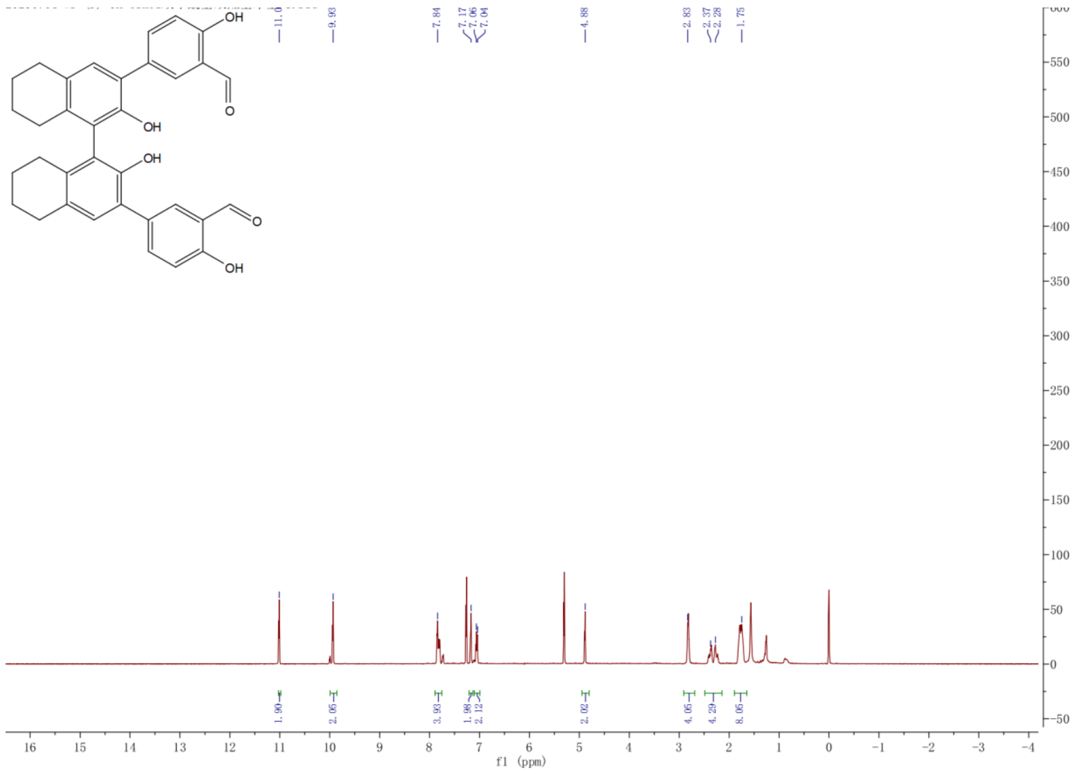

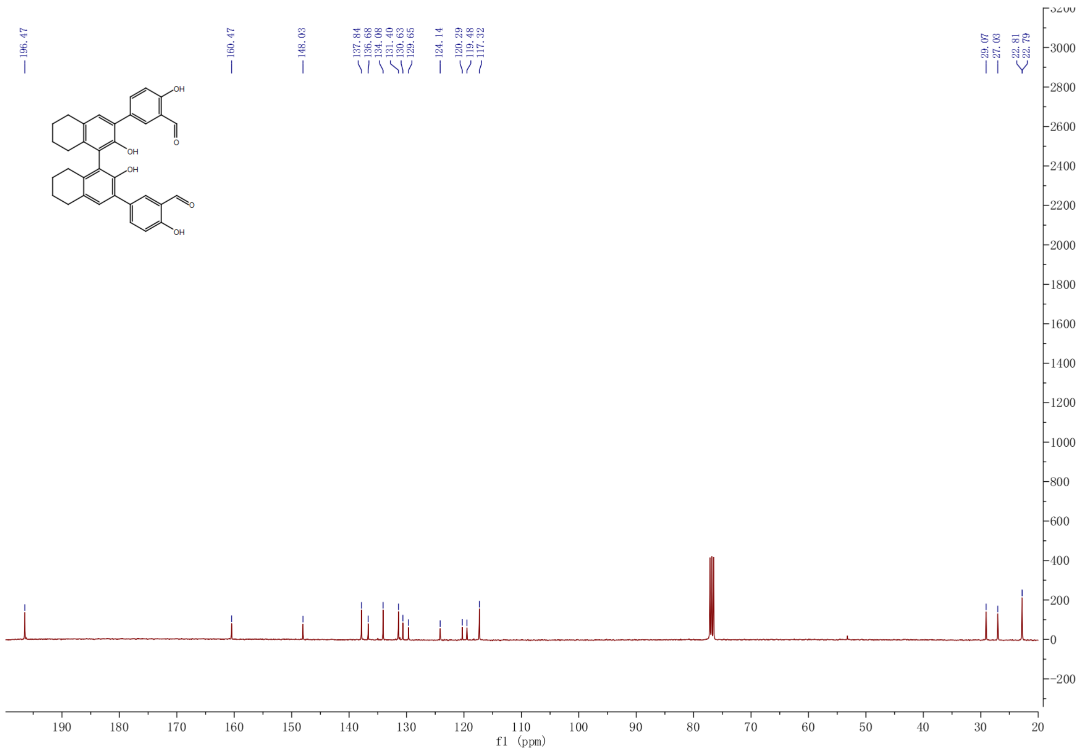

Figure S6. ^{1}H NMR, ^{13}C NMR of S-**1** ($CDCl_3$)

2.7 ^{1}H NMR of RS-4

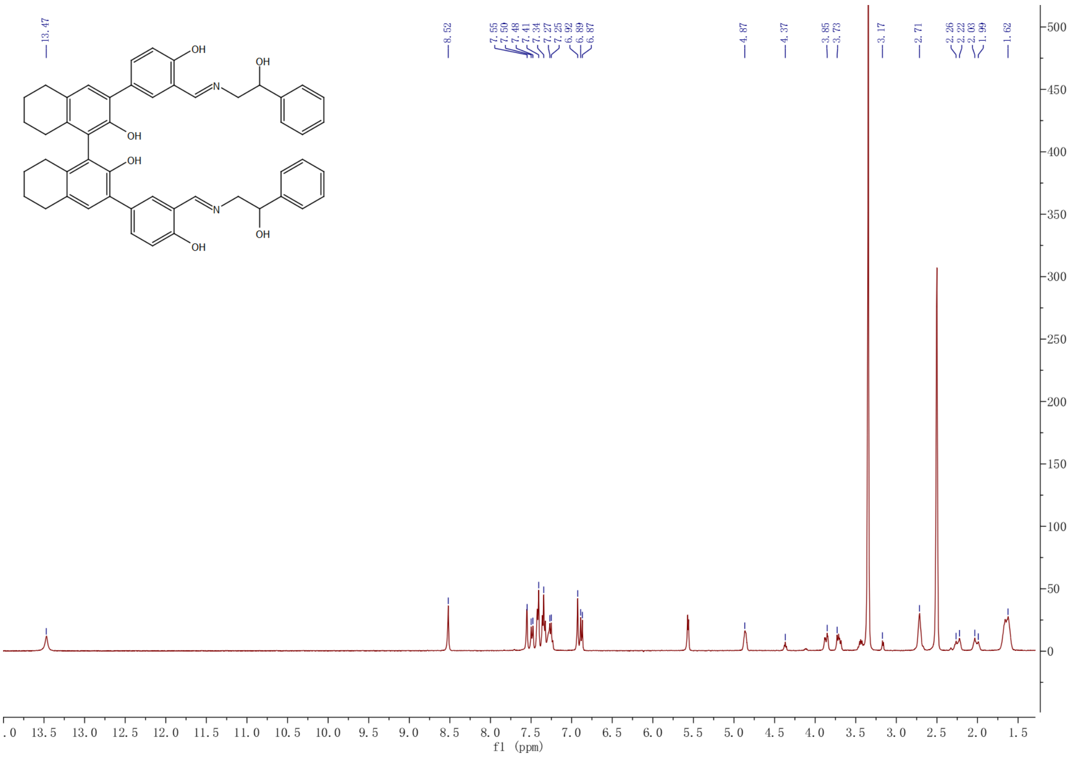

Figure S7. ^{1}H NMR of RS-**4** ($CDCl_3$)

3. Fluorescence spectras of R-2

Figure S8a. shows that the fluorescence intensity of probe R-**2** in methanol solution was relatively weak at λ_{exc} = 270 nm, but when 100 equivalents of S-2-amino-1-phenylethanol were added into the probe, it exhibited a significant enhancement of the fluorescence intensity, increasing from 1467 to 3096, which is an increase of 2.1 times. In contrast, 100 equivalents of R-2-amino-1-phenylethanol had no significant fluorescence response, indicating that R-**2** had different fluorescence responses between the two configurations of 2-amino-1-phenylethanol.

To explore further the behavior of the probe with the increase of the concentration of 2-amino-1-phenylethanol, a fluorescence titration experiment was carried out. Figure S8b. shows the changes in fluorescence intensity of R-**2** as S-2-amino-1-phenylethanol was added in steps from 10 equivalents to 100 equivalents. As the concentration of S-2-amino-1-phenylethanol increased, the fluorescence intensity of R-**2** also increased continuously, increasing from the initial intensity of 1185 to a maximum of 3456. However, the fluorescence intensity of R-2-amino-1-phenylethanol did not change significantly with the increase of the equivalent added, remaining approximately constant at around 1000.

The results of the fluorescence titration experiment are further explored by plotting the change in fluorescence intensity of S-2-amino-1-phenylethanol and R-**2** as a function of the equivalent added, as shown in Figure 1d. This figure shows that the fluorescence intensity of S-2-amino-1-phenylethanol increased steadily with the addition of equivalent, showing a good linear relationship with a correlation coefficient of R=0.9946, indicating that the probe R-**2** has a high affinity for S-2-amino-1-phenylethanol and that it can detect very low concentrations of this compound.

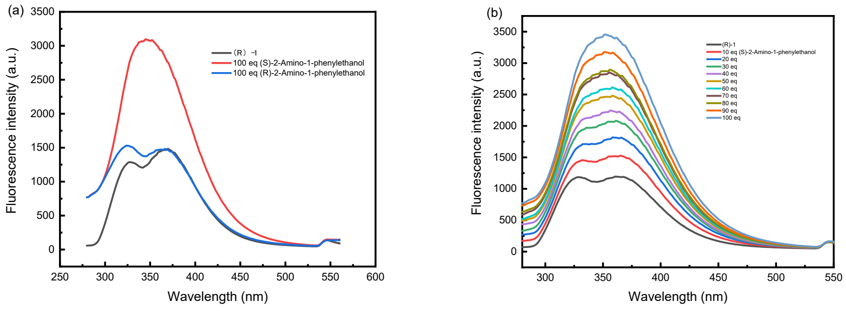

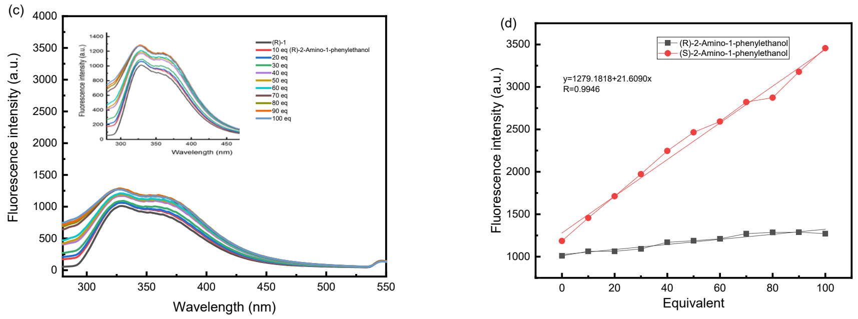

Figure S8. (**a**) Fluorescence spectrum of R-**2** ($2.0*10^{-5}$ M) for 100 eq of S-2-amino-1-phenylethanol (0.1 M) and R-2-amino-1-phenylethanol (0.1 M) in methanol solution; (**b**) Fluorescence titration spectrum of R-**2** ($2.0*10^{-5}$ M) for S-2-amino-1-phenylethanol (0.1 M) in methanol solution (0-100 eq); (**c**) Fluorescence titration spectrum of R-**2** ($2.0*10^{-5}$ M) for R-2-amino-1-phenylethanol (0.1 M) in methanol solution (0-100 eq); (**d**) Trends of R-**2** ($2.0*10^{-5}$ M) fluorescence intensity after the addition of different equivalent 2-amino-1-phenylethanol (0.1 M). (λ_{exc} = 270 nm, slits = 2.5/2.5 nm)

To explore the enantiomeric composition of 2-amino-1-phenylethanol, fluorescence intensity changes was observed by mixing R-**2** with 10 eq 2-amino-1-phenylethanol under different ee value at λ_{exc} = 270 nm (ee = [S]-[R]/[S]+[R]), it was found that with the increase of ee, the fluorescence intensity increases slowly, and obviously presents a linear relationship, according Figure S9 can be used to determine the enantiomer composition of 2-amino-1-phenylethanol through the probe.

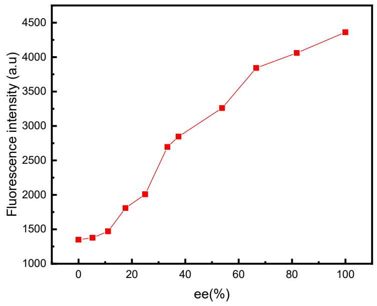

Figure S9. Fluorescence intensity change trends of R-**2** ($2.0*10^{-5}$ M) toward 2-amino-1-phenylethanol at virous ee values in methanol solution. (λ_{exc} = 270 nm, slits = 2.5/2.5 nm)

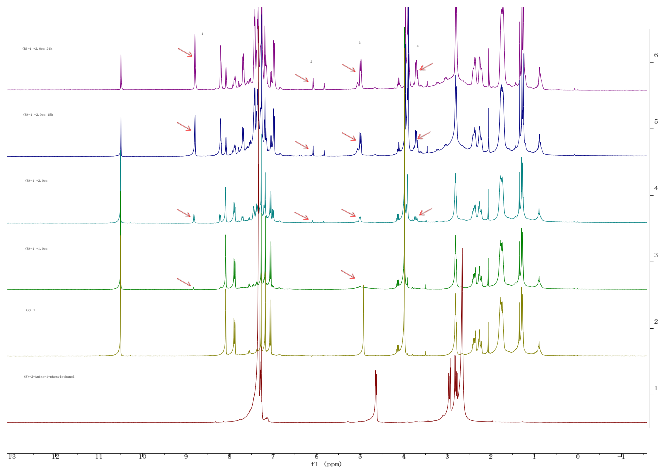

Figure S10. ^{1}H NMR spectra for the reaction of R-**2** with S-2-amino-1-phenylethanol (1.0-2.0 eq) in $CDCl_3$ solution.

In order to investigate the recognition mechanism of the probe with S-2-amino-1-phenylethanol and the reason for the fluorescence enhancement, ^{1}H NMR titration experiments were performed. $CDCl_3$ was used as solvent for both the probe and S-2-amino-1-phenylethanol, and some changes in the NMR titration spectra were observed with the addition of 1.0 eq and 2.0 eq of aminoethanol to R-**2**. However, the changes were not obvious and new peaks were roughly observed, which may be due to the reaction of the probe with aminoethanol but the reaction time was not enough or the equivalent amount of S-2-amino-1-phenylethanol was not enough. Thus, kept the system at room temperature for 15 h and 24 h, the NMR spectrogram was obviously found that there were significant new peaks, we made new assignments for these new peaks. It was observed that the aldehyde group peak at 10.5 ppm in the probe was weakened and an obvious single peak was generated at 8.8 ppm, which might be due to the condensation reaction between the aldehyde group and the amino group in S-2-amino-1-phenylethanol, which generated the -CH=N-, and a single peak was also generated at 6.08 ppm, this should be the signal peak of hydrogen in -OH group on amino alcohol, the signal peak at 5.0 ppm may be the hydrogen on the carbon connected to the benzene ring in amino alcohol, and the hydrogen at 3.71 ppm should be the hydrogen on the $-CH_2$ connected to the amino group.

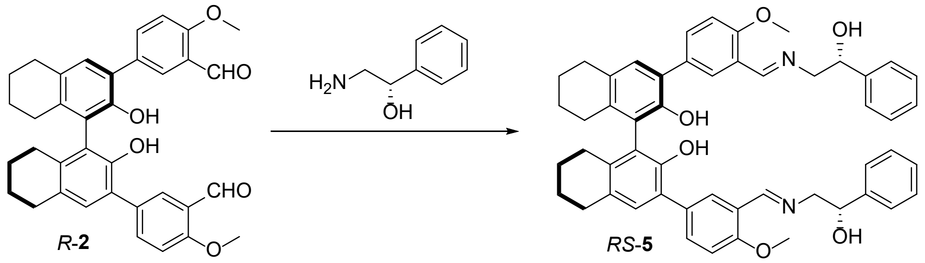

According to NMR spectrum, the probe did react with *S*-amino-1-phenylethanol, but it was impossible to determine whether a single substituted compound or a double substituted compound was formed based on this information, a mass spectrometry experiment was conducted in the methanol, the R-**2** with an excessive amount of S-2-amino-1-phenylethanol and kept the mixture at room temperature for 24 hours, according to the mass spectrum data, R-**2** reacted with S-2-amino-1-phenylethanol formed a disubstituted compound RS-**5**, as shown in Figure S11, a significant peak at m/z = 801.3387 was observed for the RS-**5** (calcd for [M+H]: 801.3898).

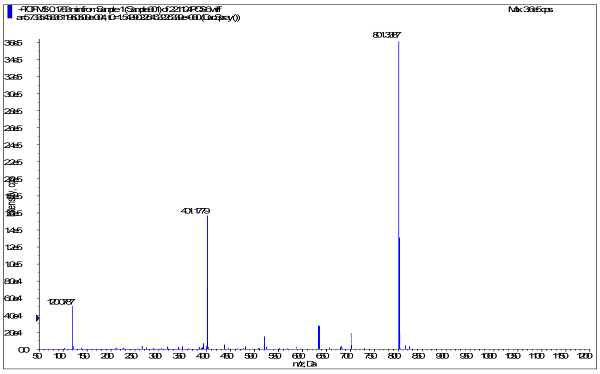

Figure S11. The mass spectrum of the mixture of R-**2** and S-2-amino-1-phenylethanol in methanol solution.

4. Comparison of IR, UV and fluorescence calculated data of R-1 with experimental data

We used the same calculation method to obtain the following calculated UV fluorescence and IR data. Fig. S12 Compare with Fig. S12, the maximum absorption wavelength for UV calculation is 270 nm, while the actual test result is 250 nm. Fig. S13 and Fig. S14 show the IR and fluorescence comparisons, respectively. The maximum emission wavelength of fluorescence in the experimental test is 335 nm, while the calculated result is 350 nm. Therefore, the calculated result is roughly the same as the actual experimental result, so the calculation strategy is realistic and reliable.

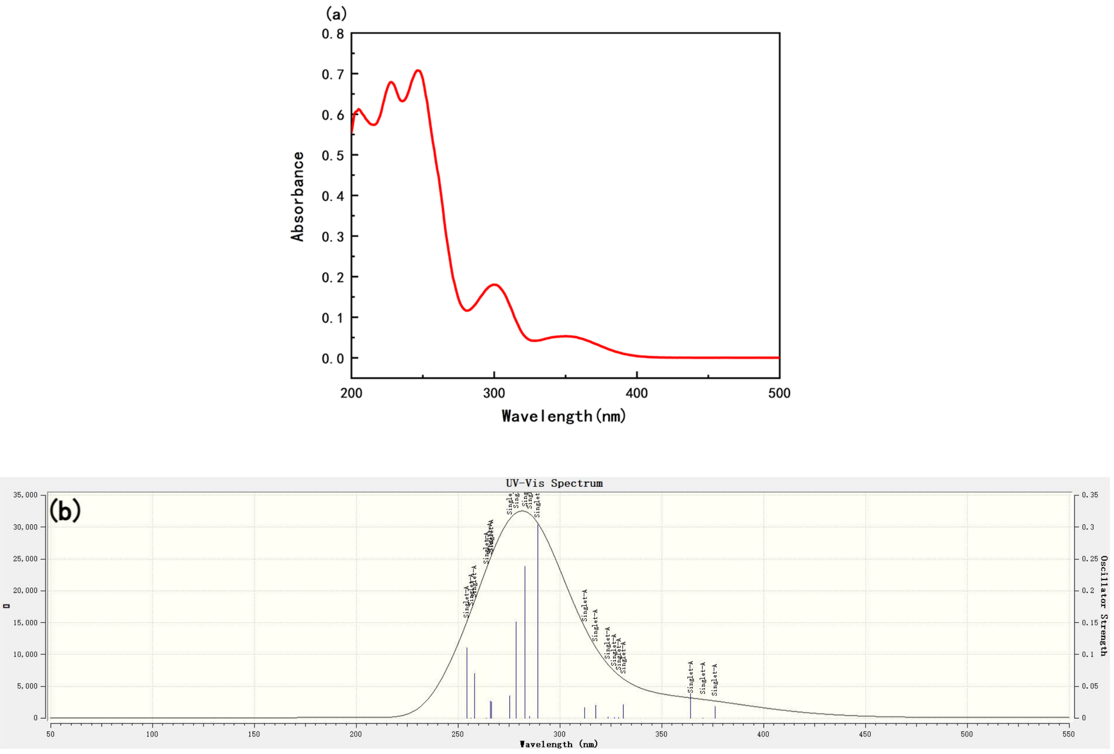

Figure S12. (**a**) UV absorption experimental data of R-**1** ($1.0*10^{-5}$ M). (**b**) Calculated UV absorption data for R-**1**.

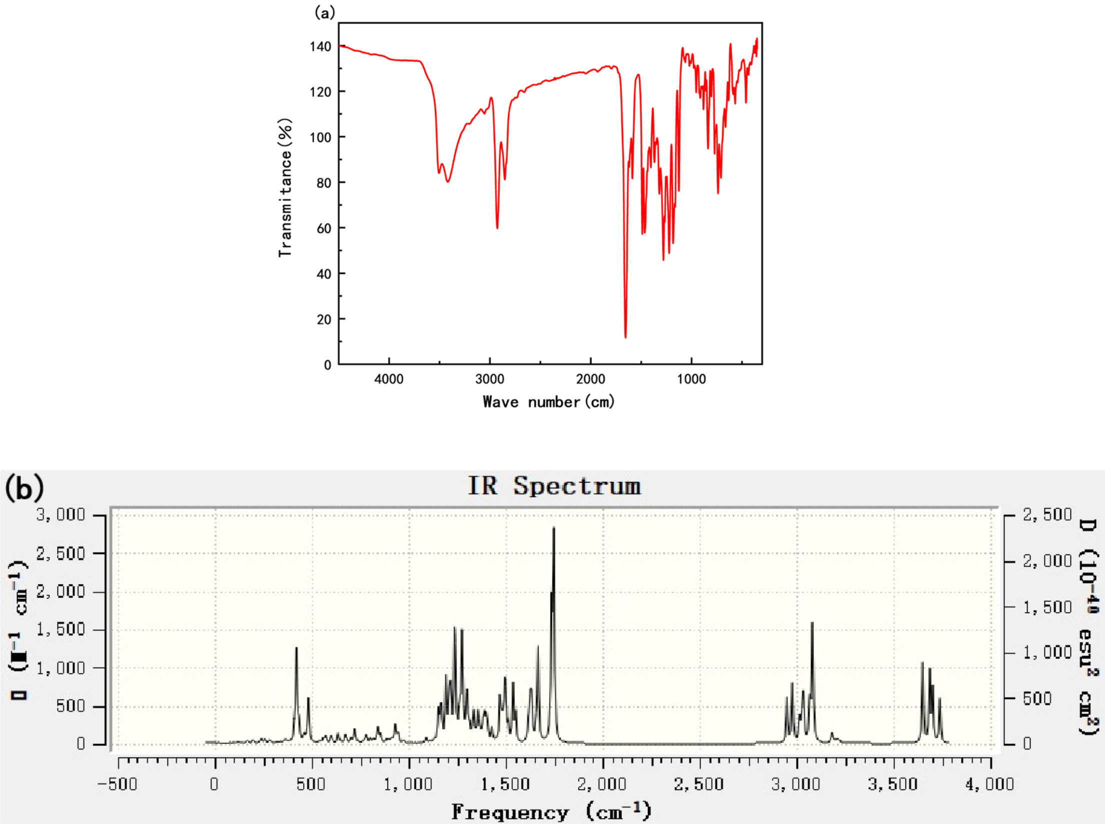

Figure S13. (**a**) IR experimental data of R-**1**. (**b**) Calculated UV IR data for R-**1**.

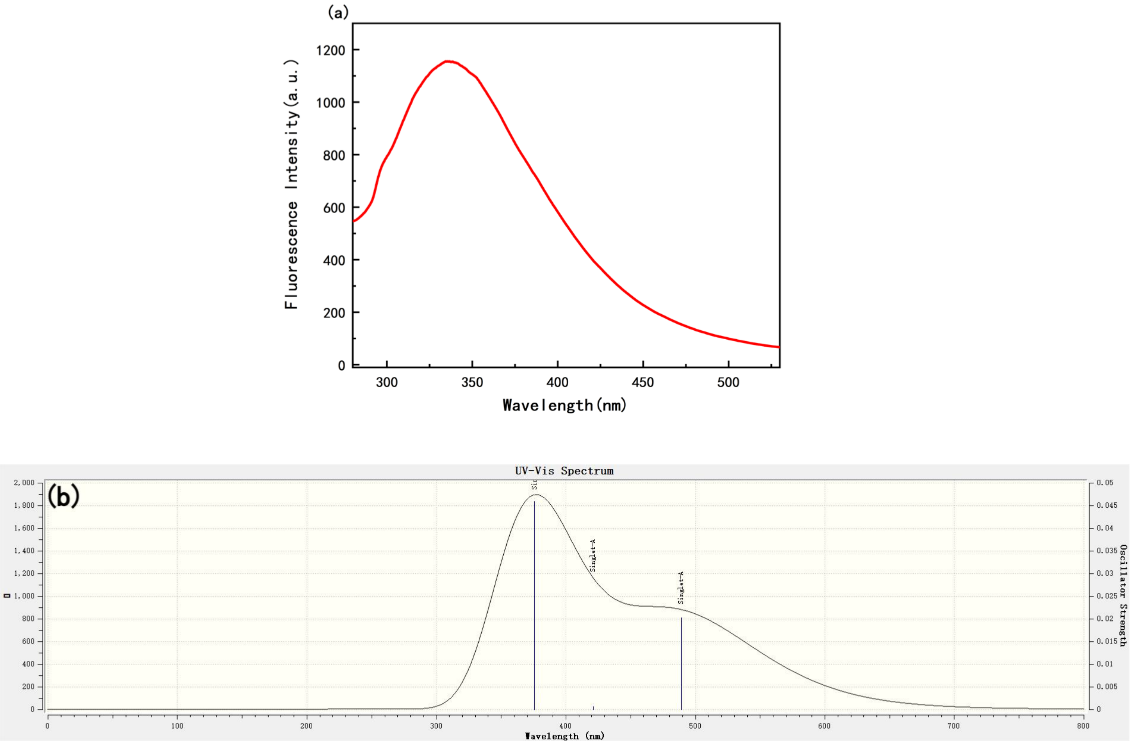

Figure S14. (**a**) Fluorescence experimental data of R-**1** (1.0*10^{-5} M). (**b**) Calculated fluorescence data for R-**1**.

5. Specific data for electron-hole analysis

Integral of hole: 1.000013
Integral of electron: 0.999903
Integral of transition density: 0.000016
Transition dipole moment in X/Y/Z: -0.004586 -0.079263 -0.016237 a.u.
Sm index (integral of Sm function): 0.00825 a.u.
Sr index (integral of Sr function): 0.08134 a.u.
Centroid of hole in X/Y/Z: -2.314877 -0.929466 1.362993 Angstrom
Centroid of electron in X/Y/Z: -6.660559 1.765986 -1.574813 Angstrom
D_x: 4.346 D_y: 2.695 D_z: 2.938 D index: 5.898 Angstrom
Variation of dipole moment with respect to ground state:
X: 8.211801 Y: -5.093451 Z: 5.551413 Norm: 11.144286 a.u.
RMSD of hole in X/Y/Z: 1.439 1.472 2.115 Norm: 2.952 Angstrom
RMSD of electron in X/Y/Z: 1.126 1.284 1.482 Norm: 2.261 Angstrom
Difference between RMSD of hole and electron (delta sigma):
X: -0.313 Y: -0.188 Z: -0.634 Overall: -0.690 Angstrom
H_x: 1.282 H_y: 1.378 H_z: 1.799 H_CT: 1.446 H index: 2.606 Angstrom
t index: 4.451 Angstrom
Hole delocalization index (HDI): 6.72
Electron delocalization index (EDI): 10.59
Ghost-hunter index: 0.681 eV, 1st term: 3.123 eV, 2nd term: 2.442 eV
Excitation energy of this state: 2.644 eV
